# Introduction: advances in small carnivore ecology

**DOI:** 10.1093/jmammal/gyag005

**Published:** 2026-03-04

**Authors:** Aliza le Roux, Géraldine Veron, Emmanuel Do Linh San

**Affiliations:** Department of Zoology and Entomology, University of the Free State-Qwaqwa Campus, Private Bag X13, Phuthaditjhaba, Free State, 9866, South Africa; Institut de Systématique, Evolution, Biodiversité (ISYEB), Muséum national d’Histoire naturelle, CNRS, Sorbonne Université, EPHE-PSL, Université des Antilles, CP 51, 57 rue Cuvier, 75231 Paris Cedex 05, France; Department of Biological and Agricultural Sciences, Sol Plaatje University, Private Bag X5008, Kimberley, Northern Cape, 8300, South Africa

Central to most terrestrial food webs, small carnivores (Carnivora <21.5 kg adult weight; Carbone et al. 1999; Do Linh San et al. 2022) are nonetheless largely ignored by ecological researchers. Reviews (Brooke et al. 2014; Do Linh San et al. 2022) have shown how the research focus of carnivore ecologists has been the large and charismatic apex predators rather than the more numerous and highly diverse group of carnivores that typically occupy lower trophic levels. This collection showcases some of the novel and critical research currently being carried out on small carnivores, stemming from a symposium on Small Carnivores at the 13th International Mammalogical Congress that was held in Alaska, United States, in July 2023. The papers span Africa (South Africa, Tanzania, Tunisia), Asia (Philippines), South America (Brazil), and North America. This geographic coverage addresses a key weakness in the field, namely a historical research bias towards Europe and North America (Do Linh San et al. 2022). In a “publish or perish” era and a time when animal welfare in animal research is becoming increasingly scrutinized, the authors highlight the value of long-term, “slow” research and demonstrate that numerous non-invasive methods can be successfully used to study the fascinating ecology and behavior of these often-hidden mammals.

Strikingly, none of the studies in this collection focus on obligate group-living species. Crab-eating foxes (*Cerdocyon thous*), bat-eared foxes (*Otocyon megalotis*), and golden jackals (*Canis aureus*; now often reclassified as the African Wolf (*Canis lupaster*)), featured in this collection, have flexible social units in which mated pairs grow into small family groups from which young typically disperse within a year if breeding territories are available. Of these 3 facultatively social species, only the Golden Jackal is considered to be a cooperative breeder because young jackals often delay reproduction to assist their parents with raising the next year’s offspring. Fishers (*Pekania pennanti*) are largely solitary, and what we know of the small carnivores in the Philippines suggests that they also range from solitary to flexible. This social organization is typical of small carnivores, who are far less likely than large carnivores to live in permanent social groups. Taking the classification of Dalerum (2007) as a basis, 23% of large terrestrial carnivores are obligate social species, compared to only 11% of small terrestrial carnivores. Thirty-four percent of small carnivores have flexible social organizations—11% more than those found in large carnivores. This distribution opens numerous doors to the study of social behavior in carnivores but also presents several obstacles. Perhaps unsurprisingly, most well-studied small carnivores, including Meerkat (*Suricata suricatta*), Banded Mongoose (*Mungos mungo*), and Dwarf Mongoose (*Helogale parvula*), are group-living species, while the study of solitary, pair-living, and flexible species lags far behind. One of the more obvious reasons for this disparity is that data collection is far slower for species that do not live in large social groups: Moehlman and Hofer’s study (this collection) of reproductive concessions and decisions in golden jackals is based on 36 litters studied over a 14-yr period, in which only 15 of those litters had subadults remaining behind as potential “helpers.” These data stand in contrast to, for example, research on habituated meerkats (Clutton-Brock et al. 1998), in which helpers assisted 26 litters born over a 5-yr period. These litters with helpers were born from a total of 57 breeding attempts monitored in that same period. It is, therefore, no surprise that most of our theorizing on the drivers of cooperative behavior is based on data gleaned from highly social species, which skews our perceptions and assumptions. This is an undoubtedly important oversight, as the ancestral condition for mammalian social organization appears to be solitary (Lukas and Clutton-Brock 2013).

Small carnivores are excellent model systems with which to examine the drivers and consequences of monogamy as well as biparental care. For example, Moehlman and Hofer used a reproductive skew model built on 7 yrs of behavioral data to untangle the drivers of helping behavior in golden jackals, showing that selection pressure is stronger on dominant breeders (the monogamous pair) to retain helpers than on helpers to disperse and breed. Monogamy—with very limited polygamy or serial monogamy—is also common in bat-eared foxes (*Otocyon megalotis*; Lamprecht 1979; Maas and Macdonald 2004), and is underpinned not by helping behavior but by particularly heavy paternal investment in raising offspring. In this collection, le Roux and colleagues begin to uncover the endocrinology of this parental care system in wild bat-eared foxes. Their findings, that pair bonding and biparental care lead to an apparent reduction in circulating glucocorticoids, suggest that monogamy may have physiological benefits beyond the evident fitness benefits of raising offspring with a mate. In obligate social species, additional social pressures related to dominance rank and competition could confound investigations into monogamy and the biparental care that is often seen in monogamous pairs. Paternal care is more common in carnivores than other mammals—32% of carnivores vs 10% of mammalian species (Kleiman and Malcolm 1981; de Bruin et al. 2016)—with >80% of the carnivores exhibiting paternal care being small carnivores. This pattern suggests that small carnivores, in particular, are an exceptional group in which to study not only social evolution, but the intricacies of mammalian fatherhood. Studying flexible, pair-living and solitary species could be key to unraveling the evolution of social behavior, cognition, communication and, most importantly, the limits and benefits of behavioral plasticity in the face of Global Change (Marneweck et al. 2022; Do Linh San 2024; Valdez et al. 2025).

Small carnivores are remarkably flexible in adapting their behavior to environmental changes, to a larger extent than most large and apex predators do (Do Linh San 2024). A further theme that is therefore explored well in this collection is the behavioral plasticity of small carnivores in response to their ecological (not social) environment, described through data obtained from fecal samples, camera traps, live traps, and citizen-science approaches. Live traps and camera traps are widespread methods of studying wildlife ecology (Miranda Paez et al. 2021; Pardo et al. 2021), and can be used particularly well in the study of elusive small carnivores, if designed appropriately. De Santana et al. (this collection) used 4 yrs of camera-trapping data to describe changes in Crab-Eating Fox activity patterns, showing a dip in activity during summer months. Fewer detections during full-moon nights, when ambient light levels were at their highest, indicate a behavioral pattern that contrasts sharply with the heightened activity (Botha et al. 2022) and reduced risk perception (Welch et al. 2017) of some other small carnivores on full-moon nights. Similar to de Santana’s findings, though, ocelots (*Leopardus pardalis*; Sergeyev et al. 2023) and Iberian lynxes (*Lynx pardinus*; Penteriani et al. 2013) moved less during full-moon nights. Mesopredator responses to ambient light are complex, as they need to both find prey and avoid becoming prey: during bright lunar phases, their prey becomes more visible—but therefore may hide or reduce activity (Pratas-Santiago et al. 2017; Oosthuizen et al. 2025), as do the small carnivores themselves. In the dark, the benefit of reduced exposure to visual hunters needs to be weighed against the reduced visibility of prey. Therefore, senses other than vision—using acoustic (Renda and le Roux 2017) and olfactory cues (Hughes et al. 2010)—may be particularly important to small, nocturnal carnivores, as they balance the risks and rewards of changes in ambient light. This kind of adaptive behavioral plasticity in the sensory realm also translates into the way that small carnivores respond dynamically to changes in the social environment: for example, Yellow Mongoose (*Cynictis penicillata*) exhibit excellent awareness of their changing social environment while foraging, and switch between acoustic and visual alarm signals, depending on context (le Roux et al. 2008). This kind of multimodal responsiveness, and the neurological architecture underlying such flexibility, has hardly received attention in the literature but may well be common in small carnivores.

The plasticity of small carnivore hunting behaviors is also evident in Shively et al.’s work on fishers (this collection). Dietary variation is common amongst small carnivores, but the limits of flexible foraging behavior are rarely investigated. Shively and colleagues used a novel metabarcoding approach to study diet composition in 2 reintroduced populations of fishers, not only validating the method as appropriate for showing relative contribution of different prey items, but also showing its inherent value in planning for conservation of rare species. The authors clearly demonstrated how the limited availability of a preferred prey item can curb the growth of reintroduced small carnivores, who have limited options for meeting their dietary requirements. Their metabarcoding approach has advantages over other techniques for the analyses of fecal data, such as isotope analyses (Botha and le Roux 2022) or the visual inspection of fecal contents (Jumbam et al. 2019; Plaatjie et al. 2024), and the authors showed how working closely with zoos can enhance and complement field-based ecological research.

In this collection, Hayder, Fernandez et al. highlight perhaps the most fundamental challenge in our understanding of small carnivores: the basic distribution and population ecology of many species remain unknown. In their review of the small carnivores of the Philippine islands, Fernandez et al. discovered only 68 articles written over a 34-yr span. In describing the detailed life history and population ecology information available for these species, they demonstrate how the dearth of knowledge undermines conservation efforts, particularly in island habitats. This challenge is not unique to islands, however, as Hayder et al. show in their in-depth investigation of the distribution of the Saharan Striped Polecat (*Poecilictis libyca*) in Tunisia. This thorough study combined multiple data sources, including museum, online repository, and spotlighting records, as well as interviews with local communities, to construct a detailed, up-to-date distribution map of polecats in Tunisia. Considering that small carnivores—unlike large carnivores—are likely to adapt well to land-use changes associated with Global Change (Clements et al. 2024), it is crucial that we put more effort towards understanding the basic population ecology of small carnivores.

An important thread that runs through most articles in this collection is how humans interact with small carnivores, whether intentionally or not. The Saharan Striped Polecat is valued, for example, for its perceived medicinal properties—a fact that could, hypothetically, be used to help bolster conservation efforts if this incentivizes sustainable harvesting by local communities. Carnivores feature prominently in human uses of natural resources. For example, Williams et al. (2025) have shown that carnivores, including small carnivores, are commonly used in cultural practices such as traditional attire and consumption. However, the negative perception of small carnivores as livestock hunters remains prominent (e.g., Logan et al. 2014; Somers et al. 2018) and therefore counteractive to conservation efforts. Emphasizing the ecosystem services performed by small carnivores (Williams et al. 2018; Nakashima and Do Linh San 2022) has the potential of boosting conservation efforts in communities that visibly co-exist with these species, but the efficacy of such an approach remains to be explored.

Collectively, the articles in this special feature not only fill critical knowledge gaps for specific species and regions but also illuminate a path forward for the field of small carnivore ecology by focusing on 4 key strategies: (i) integrate diverse methods; (ii) move beyond well-studied species and regions; (iii) focus on multi-species and multi-trophic interactions; and (iv) bridge the research-conservation gap. Indeed, progress hinges on an integrated approach by combining powerful new technologies like DNA metabarcoding and advanced camera-trap analyses with invaluable, long-term behavioral studies and the inclusion of local ecological knowledge. As recently highlighted by Do Linh San et al. (2022), future research must continue to shift focus towards understudied species and ecosystems to build a truly global understanding of small carnivore ecology. Creative approaches, such as camera traps enclosed within a box housing a small tunnel, will go a long way towards including species that we overlook or miss with traditional approaches, as recently demonstrated in Europe and Africa (Davis et al. 2025; Granata et al. 2025; Otte et al. 2025). Small carnivores offer opportunities to study fundamental questions about the proximate and ultimate drivers of mammalian behavior, going beyond our current focus on obligate social, often larger, species. By investigating community-level interactions (Mills et al. 2019; Begg et al. 2022) and explicitly linking ecological findings to management plans and conservation strategies (Nakashima et al. 2022), researchers can ensure that these diverse and functionally vital species are no longer overlooked in a rapidly changing world.
